# Early Changes and Indicators Characterizing Lung Aging in Neonatal Chronic Lung Disease

**DOI:** 10.3389/fmed.2021.665152

**Published:** 2021-05-31

**Authors:** Jennifer Sucre, Lena Haist, Charlotte E. Bolton, Anne Hilgendorff

**Affiliations:** ^1^Mildred Stahlman Division of Neonatology, Department of Pediatrics, Vanderbilt University, Nashville, TN, United States; ^2^Institute for Lung Biology and Disease and Comprehensive Pneumology Center With the CPC-M bioArchive, Helmholtz Center Munich, Member of the German Center for Lung Research (DZL), Munich, Germany; ^3^Center for Comprehensive Developmental Care (CDeCLMU), University Hospital Ludwig-Maximilian University, Munich, Germany; ^4^Division of Respiratory Medicine, NIHR Nottingham Biomedical Research Centre, School of Medicine, University of Nottingham, City Hospital NUH Campus, Nottingham, United Kingdom

**Keywords:** neonatal chronic lung disease, bronchopulmonary dysplasia, lung aging, inflammation, mechanical ventilation, oxygen toxicity, lung function, preterm infant

## Abstract

Infants suffering from neonatal chronic lung disease, i.e., bronchopulmonary dysplasia, are facing long-term consequences determined by individual genetic background, presence of infections, and postnatal treatment strategies such as mechanical ventilation and oxygen toxicity. The adverse effects provoked by these measures include inflammatory processes, oxidative stress, altered growth factor signaling, and remodeling of the extracellular matrix. Both, acute and long-term consequences are determined by the capacity of the immature lung to respond to the challenges outlined above. The subsequent impairment of lung growth translates into an altered trajectory of lung function later in life. Here, knowledge about second and third hit events provoked through environmental insults are of specific importance when advocating lifestyle recommendations to this patient population. A profound exchange between the different health care professionals involved is urgently needed and needs to consider disease origin while future monitoring and treatment strategies are developed.

## Disease Characteristics and Predispositions

As the most common chronic lung disease in infants, Bronchopulmonary Dysplasia (BPD) is associated with long-term sequelae that persist into adulthood (1, 2). Despite significant improvements in perinatal care, i.e., surfactant and antenatal corticosteroid treatment together with improved ventilation strategies, the incidence of BPD has remained unchanged or even increased amongst the most immature infants (3). This is presumably due to a significant reduction of mortality rates together with an increase in the overall number of treated infants born significantly premature. The varying incidence of BPD between newborn care centers closely reflects differences in patient population and infant management practices (4–7). Recent publications report an incidence of BPD of up to 68% in very low birth weight infants (401–1,500 g) born prior to 29 weeks of gestation, or up to 77% in infants born at <32 weeks of gestation with a birth weight below 1 kg (5, 8, 9), with numbers predominantly derived from high-income countries.

The disease is classified into three different severity grades (mild, moderate, severe) according to the need for supplemental oxygen and/or ventilator support for >28 days of life, or beyond 36 weeks postmenstrual age (PMA) (2). Environmental insults associated with preterm birth sum up to sustained inflammation and extensive matrix remodeling resulting in substantial changes to the scaffold provided for the developing organ in concert with functional abnormalities as a consequence of diffuse fibrotic changes and increased smooth muscle hypertrophy in small pulmonary arteries and airways (10). The characteristic histopathologic changes of impaired alveolarization and vasculogenesis (2) are clinically mirrored by signs of impaired respiratory gas exchange, i.e., alveolar hypoventilation with resultant hypercapnia and hypoxemia leading to a mismatch of ventilation and perfusion (11).

Large clinical trials have identified numerous risk factors for the development of BPD, including congenital and nosocomial infections, mechanical ventilation, and oxygen toxicity (12–17). Poor nutritional support, vitamin deficiency as well as insufficient adrenal and thyroid hormone release in the very premature infant further increase the risk after birth (18–20). Prenatal risk factors influence the capacity of the developing lung to respond to these injuries. Preeclampsia is known as an independent risk factor not only for preterm delivery, but more importantly also for BPD, despite its underlying molecular mechanisms remaining elusive (21, 22). Intrauterine growth retardation increases the risk of BPD 3- to 4-fold (23–27), most likely through their impact on altered growth factor signaling and subsequently impaired alveolar and vascular development (28). Exposure to prenatal smoke, although largely underestimated, has been shown to significantly contribute to disease development, potentially beyond growth restriction (29, 30), and even the prenatal application of established therapeutic measures has to be critically reviewed. Here, antenatal betamethasone, despite its broad prenatal application to enhance lung maturation and to prevent respiratory distress while reducing BPD rates (31, 32), has been shown to increase indicators of lipid membrane peroxidation (33). This word of caution is in line with observations on behalf of postnatal dexamethasone treatment, where adverse effects on cardiac function, life expectancy, and neurologic development have been observed (34, 35). The broad use of antenatal maternal antibiotic treatment on the other hand not only significantly affects the bacterial flora of the child (36) but leads to sustained alterations of immune functions, e.g., in the response of invariant natural killer T cells, as indicated by studies in mice (37). Although prenatal exposure to smoke and antibiotics were shown to provoke lung changes on the molecular level as shown by studies in mice, further investigations are needed in order to establish these risk factors clinically, as prior published data from preterm infants are inconsistent (38, 39).

With regard to the genetic background impacting on the clinical course, prior work demonstrates that gene polymorphisms account for 53% of the variance in BPD (40). Identified genetic abnormalities include mutations in genes associated with surfactant biogenesis, innate immune response (41, 42), and superoxide dismutase (43), with details of the possible pathophysiology explained in the paragraphs below. The higher risk for the development of BPD and pulmonary arterial hypertension (PAH) in male preterm infants (44) has been associated with differences in hormonal regulation (45), although longer term, females with a history of BPD seem to be more severely affected (46).

## From Cause to Consequence: Biological Properties, Pulmonary Structure, and Lung Function

### Inflammation and Oxidative Stress Response

The sustained inflammatory processes that characterize BPD are caused by pre- as well as postnatal mechanisms with key players highlighted in the paragraph above. Both, infections as well as the corresponding immature immune response play an important role in the initiation and perpetuation of inflammatory processes characterizing and driving BPD development (14, 47–49). Perinatal processes, e.g., the fetal inflammatory response syndrome (FIRS), chorioamnionitis, or their development into congenital and nosocomial infections results in neutrophil influx into the immature lung, with an increased number of monocytes and macrophages during the so called “second wave” of inflammation (27, 50, 51). Here, specific pathogens such as Gram-negative bacteria play an important role, clearly increasing the risk for neonatal chronic lung disease (52). Later nosocomial infections are caused by a different, “non-maternal” spectrum of pathogens (e.g., Staphylococcus epidermidis, *Escherichia coli*) that are likewise associated with BPD development (53, 54).

Postnatally, non-infectious causes such as baro- and volutrauma during mechanical ventilation in concert with the effects caused by moderate or severe hyperoxia further contribute to or even initiate the inflammatory processes locally and on a systemic level (55–58). Ongoing studies addressing the debate, whether recruitment of inflammatory cells to the injured lung or activation of resident cells are primarily responsible to start and drive the vicious circle of inflammation (59–62) will give important insight for mechanistic understanding and development of targeted therapies. At the same time, extracellular matrix (ECM) remodeling itself further promotes lung inflammation through the release of proteases and inflammatory mediators such as transforming growth factor beta (63–65). The regulation of NF-kB signaling in inflammatory processes (66) and its lately discovered role in alveolo- and vasculogenesis that may even bear therapeutic potential (67, 68) demonstrate the close relation of lung inflammation during organ development to fundamental and long-lasting structural changes.

The degree of lung inflammation and ECM remodeling as well as early alveolar epithelial dysfunction is directly related to the cellular capacity to respond to postnatal environmental challenges. The relative deficiency of antioxidants and inhibitors of proteolytic enzymes render the immature lung especially vulnerable to the effects of inflammatory mediators and toxic oxygen metabolites (69–72). Different markers have been investigated to indicate enhanced oxidative stress in the preterm infant. Elevated urinary malondialdehyde concentrations in the first week of life, generated by peroxidation of lipid membranes after oxidant-mediated injury, were correlated with the risk for oxygen radical diseases including BPD (33). In a murine model, reduced superoxide dismutase 3 (SOD3) in reaction to postnatal hyperoxia was associated with alveolar injury, whereas overexpression of SOD3 attenuated hyperoxic injury in an alveolar epithelial cell line (73). Decreased pulmonary antioxidant concentrations have also been measured in the lavage of preterm infants (74). In line with this, intratracheal application of recombinant human CuZn superoxide dismutase at birth improved pulmonary outcome in high-risk premature infants at 1 year corrected age (75). Studies indicated that adolescent BPD patients have evidence of heightened oxidative stress in the airway, suggesting that long-term respiratory abnormalities after preterm birth align with sustained alterations of the oxidative stress response (76). These effects might even translate into altered responses to viral infections in later life (77).

The increased susceptibility of the developing organ to environmental challenges and the induction of long-term consequences are supported by the observation that significant maturational differences exist between neonatal and adult lung cells in response to lung injury. While chronic oxygen exposure (60% for 14 days) enhances lung vascular and airway smooth muscle contraction and reduces nitric-oxide relaxation in the neonatal rat lung, the opposite occurs in adults (78). In line with the observations obtained in neonatal mice, long-term effects of hyperoxia exposure in the first week of life (100% for 4 days) include increased mortality associated with pulmonary vascular disease and the development of right ventricular strain and PAH in mice (79). The alteration of bone morphogenic protein (BMP) signaling likely contributes to this adult lung phenotype. Other mechanisms explaining the increased susceptibility to injury and the subsequent likelihood of long-term effects observed in the newborn lung are suggested from studies by Balasubramaniam et al. showing that hyperoxia reduces bone marrow derived, circulating, and lung endothelial progenitor cells in the developing organ in contrast to adult mice (80), indicating early exhaustion of repair and regeneration capacities. The risk for long-term effects is furthermore mitigated by the affection of central processes such as cell cycle regulation, i.e., upregulation of P21 by hyperoxia exposure together with decreased histone deacetylase activity (81). The studied effects of excessive oxygen exposure on DNA methylation further contribute to the picture of accumulating damage in the face of reduced compensatory mechanisms in the immature lung (82).

The outlined injury effects ultimately result in the impairment of lung growth based on significantly imbalanced growth factor signaling. Orchestrating the interaction of the epithelial, mesenchymal, and endothelial cell compartment during the fine-tuned development of the gas exchange area, Notch and Wingless Int-1 (Wnt), the fibroblast and platelet derived growth factor (FGF, PDGF) as well as the BMP and the vascular endothelial growth factor (VEGF) play a critical role (83–89).

### Morphogenetic Changes to the Pulmonary Cellular and Extracellular Matrix

The release of cytokines such as the transforming growth factor (TGF) –β, tumor necrosis factor (TNF) alpha and interleukins, e.g., IL-1beta in response to lung inflammation and subsequent events such as ECM remodeling and cellular injury significantly contributes to the imbalance in growth factor signaling and leads to the activation of different transcription factors enhancing apoptosis in numerous cell types (90–92). The interference with these transcription factors disrupts normal lung morphogenesis (67) and drives the onset of chronic bronchial inflammation and subsequent pulmonary emphysema in the adult organ (93). In the developing organ, the altered regulation of transcription leads to long-term effects such as the impairment in alveolar and vascular development resulting from nuclear factor kappa B (NF-kB) suppression (66, 68, 94), unequivocally linking key processes in BPD development such as infection and inflammation with altered growth factor signaling and transcriptional regulation. The characteristic co-existence of defective alveolar and capillary formation is determined by impaired angiogenic growth factor signaling (67) and ultimately results in sustained vascular disease, in many cases presenting as PAH and/or impaired lung lymphatic drainage (95, 96). The typical reduction in pulmonary expression levels of VEGF and its receptors (97–99), accompanied by diminished endothelial nitric oxide synthase (eNOS) and soluble guanylate cyclase (sGC) in lung blood vessels and airways (100, 101) reflects the expression pattern observed in aged mice (102) and likely contributes to the reduced plasticity of lung capillaries (103). Treatment with recombinant human VEGF during or after hyperoxia exposure improved not only vessel growth but also alveolarization in the lungs of newborn rats (104).

Both direct effects of shear stress, oxygen toxicity, inflammation, and hormonal regulation as well as subsequent impairment of growth factor signaling alters critical events in endoderm to mesoderm transition and myofibroblast proliferation and leads to severe alterations of the pulmonary scaffold (105, 106). Increased matrix remodeling characterized by the greater abundance and abnormal distribution of elastin together with the deformation of collagen scaffolding has been demonstrated in humans and animal models (107–109). The degradation of lung elastin is indicated by e.g., increased urinary excretion of desmosine, preceded, and paralleled by increased elastase activity (110–112). Desmosine, a breakdown product of the mature elastic fiber was found to predict disease severity and outcome in adult patients with acute respiratory distress syndrome (ARDS) (113), indicating the importance of the delicate balance of proteases and their inhibitors. Complicating the definition of friend or foe in the developing organ, the presence of elastases including metalloproteinase activity is crucial as evidenced by studies showing that complete matrix-metalloproteinase deficiency promotes lung remodeling resembling BPD (114).

The significant changes to the structural integrity of the ECM not only affect its function as a scaffold for the formation of alveoli and capillaries but as well-reveal a significant memory function through defining the fate of cells populating the developing organ (115, 116). The sustained and irreversible reorganization will furthermore result in long term effects with regard to the lung's repair and regeneration capacity, its potential for immune cell interaction, thereby determining its coping with environmental challenges, exacerbation episodes and physiologic aging (117, 118).

### Short and Long-Term Pulmonary Function in Preterm Infants With BPD

With increasing survival of infants with BPD, attempts to minimize long-term pulmonary impairment (and associated neurologic complications) has become the main focus of perinatal care (119, 120). Nonetheless, respiratory symptom presentation and suboptimal lung function are manifesting in adult life, and where detected, can be misclassified as more common respiratory diagnoses such as asthma or COPD, particularly if the early life events are not known or asked about (121, 122). In many cases, respiratory disease is not detected until acute presentation or much later in life. As respiratory function serves as a good predictor of later morbidity and mortality (123), knowledge about early changes seems crucial.

After birth, early pulmonary dysfunction is characterized by diminished lung compliance, tachypnea, and increased minute ventilation resulting in increased work of breathing with or without subsequent oxygen dependency. This clinical picture can be accompanied by an increase in lung microvascular filtration pressure that may lead to interstitial pulmonary edema as shown in animal experiments (124). The increased lung vascular resistance, typically associated with impaired responsiveness to inhaled nitric oxide and other vasodilators, can progress to reversible or sustained PAH and right heart failure (95, 96). Early measurement of lung function provides prognostic information and has shown that postnatal development of severe lung disease more likely develops into chronic disease at term (125). At this time point BPD infants present with increased respiratory tract resistance and hyper-reactive airways (126), subsequently leading to frequent episodes of bronchoconstriction and cyanosis after discharge often resulting in hospital readmission. The proportion of underlying vascular disease playing a role in these clinical manifestations beyond the effects caused by the Euler-Liljestrand mechanism (hypoxic pulmonary vasoconstriction) often remains unclear as sensitive diagnostic tools are missing (127).

In the later course following discharge, infants with BPD may remain oxygen dependent for months or years, although only a minority remains oxygen dependent beyond 2 years of age (128, 129). Oxygen dependency indicates the most severe lung disease, as these infants require hospital readmission twice as often in comparison to infants without home oxygen therapy. However, even after having outgrown oxygen dependency, patients with moderate or severe BPD still require outpatient clinic visits, readmissions, and medication in up to 70% of the cases and 30% need three readmissions in the first 2 years of life (130). A major predictor for readmission due to respiratory causes or the need for subsequent mechanical ventilation is the pCO_2_ at discharge (131). After the second year of life, hospitalization rates decline (132). Related to prematurity beyond BPD status, lower respiratory tract infections resulting from respiratory syncytial virus remain the major cause for readmission amongst preterm infants (133).

In the later course of disease, BPD is a significant risk factor for persistent wheeze and the need for inhalation therapy (odds ratio 2.7 and 2.4, respectively) affecting about 20–30% of infants with BPD at 6 and 12 months of age (134, 135). Respiratory symptoms remain common at preschool and school age (128, 136). Up to 80% of preterm infants, particularly those who presented with wheezing, demonstrate airway obstruction in early childhood and adolescence, the majority of whom are symptomatic (137–139). Important data on long-term pulmonary function in BPD patients were generated by the EPICure study (140), showing significantly lower peak oxygen consumption, forced expiratory volume at 1 second (FEV_1_) and gas transfer for those born extremely premature at school age when compared to age matched controls, not considering BPD status. Mean difference in FEV_1_ sum up to a total of 600 ml when comparing infants with extreme prematurity at birth and the respective healthy controls. Significantly lower peak workload and higher respiratory rates in combination with lower tidal volumes during peak exercise and increased residual capacity in these infants may reflect the effect of hyperinflation due to airway obstruction and/or altered pulmonary chemoreceptor function, and suggest the presence of persistent airflow limitations and reductions in alveolar surface area.

In most severe cases symptoms either persist into adulthood (141) or show transient “improvement” reflected by the absence of symptoms, later resulting in the “reappearance” of disease as a consequence of lung function decline below a clinical (or individual) threshold when the disease associated reduction in lung volume and function is met by aging processes or a newly occurring mismatch of the lung-body mass ratio and/or energy expenditure. Altered lung volumes and decreased gas mixing efficiency in BPD has been confirmed by various studies, reflecting abnormalities in lung growth (142, 143), resulting in suboptimal airway function (judging from impaired FEV1 and FEV1/forced vital capacity) in young adults (144, 145), also manifesting in suboptimal exercise capacity (145). While a diverging path in lung growth during adolescence according to spirometric measures was reported by one group (144), the EPICure study revealed no catch-up of the suboptimal lung growth from 11 to 19 years in adolescents following extreme prematurity irrespective of BPD status and even showed significant impairment in all lung function parameters in 19 year old patients born extremely premature (145). Meanwhile, Vollsaeter et al. reported parallel trajectories of lung function in early adult life (146), reflecting the overall need for more robust and contemporaneous studies.

Taken together, the functional data available suggest that early lung injury in preterm infants leads to abnormalities in lung function (and immunity) in infancy and early childhood with significant pulmonary problems persisting in the most severely affected infants. The predisposition for lung function decline in adult survivors of preterm birth is suggested by data obtained in early and later adulthood with lung function measurements in infancy being an important predictor for later lung disease and the risk for (early) lung function decline. If performed during infancy and childhood, pulmonary function tests have the potential to identify individuals at risk of long-term respiratory sequelae and will help to elucidate the impact of secondary injuries, e.g., first and second hand smoke as well as viral infections on this trajectory (147). Importantly, lung function has to be interpreted in the context of the era in which BPD was diagnosed, thus taking both the underlying BPD definition as well as the standards of perinatal care into account (2, 148, 149).

## Conclusion

**Figure 1:**
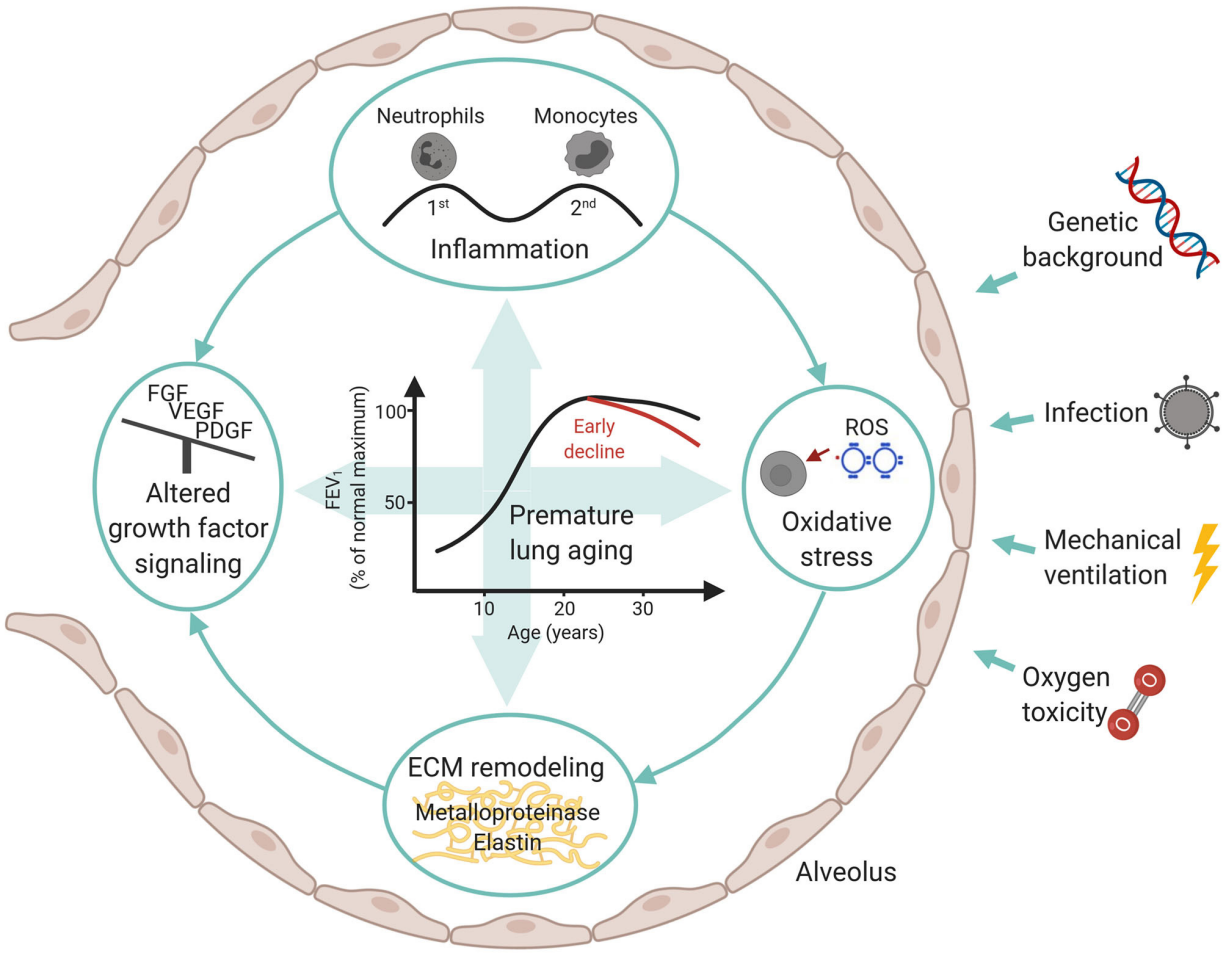
Overview over influencing factors and molecular mechanisms in BPD development with a focus on long-term consequences.

To conclude, the unique response of the developing lung to early postnatal injury is characterized by sustained inflammatory processes, ECM remodeling and a pronounced alteration in growth factor signaling that ultimately results in the characteristic histopathologic picture of impaired alveolar and vascular development. These processes are critically determined by the pulmonary capacity to respond to and compensate for environmental challenges inducing oxidative stress and imbalance in growth factor signaling (Figure 1). We now understand that the effects provoked through early organ injury lead to a characteristic response to challenges later in life and an altered lung function trajectory due to differences in the pulmonary aging process. Subsequently, treatment strategies and life-style recommendations advocated to this patient population need to acknowledge the pulmonary “memory” effects that results from early injury as well as later disease characteristics and co-morbidity development (150, 151). Therefore, pediatricians, primary care physicians and adult pulmonologists need a close and iterative knowledge exchange to adequately address these topics, including the role of second and third hits in lung function decline (152, 153).

## Author Contributions

All authors listed have made a substantial, direct and intellectual contribution to the work, and approved it for publication.

## Conflict of Interest

The authors declare that the research was conducted in the absence of any commercial or financial relationships that could be construed as a potential conflict of interest.
